# Comparing counselling models for the hazardous use of alcohol at the Swedish National Alcohol Helpline: study protocol for a randomised controlled trial

**DOI:** 10.1186/s13063-017-2005-5

**Published:** 2017-06-06

**Authors:** Eleonor Säfsten, Yvonne Forsell, Mats Ramstedt, Maria Rosaria Galanti

**Affiliations:** 1 Department of Public Health Sciences, Karolinska Institutet, 171 77 Stockholm, Sweden; 2 Centre for Epidemiology and Community Medicine, Stockholm Health Care District, 113 65 Stockholm, Sweden; 3 Department of Clinical Neuroscience, Karolinska Institutet, 171 77 Stockholm, Sweden; 4 The Swedish Council for Information on Alcohol and Other Drugs (CAN), 107 25 Stockholm, Sweden

**Keywords:** Hazardous drinking, Telephone helpline, Brief intervention, Counselling, Randomised controlled trial

## Abstract

**Background:**

Hazardous and harmful consumption of alcohol is a leading cause of preventable disease and premature deaths. Modifying the amount and pattern of risky alcohol consumption conveys substantial benefits to individuals and to society at large. Telephone helplines provide a feasible alternative to face-to-face counselling in order to increase the reach of brief interventions aiming at modifying the hazardous and harmful use of alcohol. However, there is a lack of studies on the implementation and evaluation of population-based telephone services for the prevention and treatment of alcohol misuse.

**Methods/design:**

A randomised controlled trial was designed to compare a brief, structured intervention to usual care within the Swedish National Alcohol Helpline (SAH), concerning their effectiveness on decreasing the hazardous use of alcohol. Between May 2015 and December 2017, about 300 callers are to be individually randomised with a 1:1 ratio to a brief, structured intervention (*n* = 150) or to usual care (*n* = 150). The brief, structured intervention consists of the delivery of a self-help booklet followed by one proactive call from SAH counsellors to monitor and give feedback about the client’s progression. Callers assigned to usual care receive telephone counselling according to existing practice, i.e., motivational interviewing in a tailored and client-driven combination of proactive and reactive calls. The primary outcome is defined as a change from a higher to a lower AUDIT risk-level category between baseline and follow-up. General linear modeling will be used to calculate risk ratios of the outcome events. The primary analysis will follow an intention-to-treat (ITT) approach.

**Discussion:**

The trial is designed to evaluate the effectiveness in decreasing the hazardous and harmful consumption of alcohol of a brief, structured intervention compared to usual care when delivered at the SAH. The results of the study will be used locally to improve the effectiveness of the service provided at the SAH. Additionally, they will expand the evidence base about optimal counselling models in population-based telephone services for alcohol misuse prevention and treatment.

**Trial registration:**

ISRCNT.com, ID: ISRCTN13160878. Retrospectively registered on 18 January 2016.

**Electronic supplementary material:**

The online version of this article (doi:10.1186/s13063-017-2005-5) contains supplementary material, which is available to authorized users.

## Background

The consumption of hazardous and harmful amounts of alcohol is ranked as one of the leading preventable causes of premature death and disease. In 2015, the global disease burden attributable to alcohol was quantified to 85 million disability-adjusted life years (DALYs) and the cause of 2.3 million premature deaths. Since 1990, there has only been a small decreasing trend in alcohol use, revealing a major potential for interventions to reduce alcohol as a risk factor [1]. In 2015, 15% of the adult Swedish population were consumers of hazardous or harmful amounts of alcohol [2], corresponding to approximately 1.2 million individuals. In addition, the related societal costs of problem drinking are substantial, estimated at SEK20.3 billion (2002 prices) [3]. Consequently, these patterns of alcohol consumption constitute a key risk factor for morbidity in the Swedish population, calling for effective interventions.

Most individuals who consume hazardous amounts of alcohol or have alcohol dependence never seek health care [4], and a majority succeed in modifying their alcohol-related hazardous behaviour without professional support [5]. Although brief interventions (BI) in primary care settings are effective in reducing the consumption of hazardous and harmful amounts of alcohol [6, 7], their reach is limited since systematic recognition of problematic drinking is not pursued in most health care settings [8]. Because of their easy access, telephone services provide an ideal setting for the delivery of BI to consumers of hazardous amounts of alcohol.

Population-based quit lines are an established counselling setting in smoking cessation [9] and are readily available at low cost [10]. Although national or community alcohol helplines are established in several countries (e.g., New Zealand, the US, UK and Brazil), the evidence that the support provided by such a telephone service effectively modifies alcohol consumption remains scarce [11, 12]. In fact, to our knowledge, only one study has been published on population-based telephone counselling among persons with severe problems of alcohol abuse. The intervention was seemingly effective in promoting abstinence from alcohol. However, the study had methodological problems, especially an attrition close to 77% [11].

Apart from this example, telephone services for alcohol misuse have primarily been used within multicomponent interventions and/or interventions dedicated to clinical populations [13–17]. However, telephone services targeting consumers of hazardous amounts of alcohol in the general population could constitute an efficient complement to traditional health care, potentially reducing the barriers to treatment-seeking, for instance, by allowing protected identity of the user.

The Swedish National Alcohol Helpline (SAH) was established in 2007 in order to provide directly accessible toll-free counselling for users of hazardous and harmful amounts of alcohol. This setting offers the unique possibility of evaluating the effectiveness of telephone counselling open to the general population. An observational study of the SAH clients reported substantial improvements in the use of hazardous amounts of alcohol and in mental health at 12 months [12]. However, due to lack of a control group, no robust conclusions could be drawn on the effectiveness of the counselling.

Due to the pioneer stage of telephone-based services for alcohol misuse there is also a lack of knowledge on optimal duration, content, effectiveness and cost-effectiveness of the counselling provided. Studies suggest that the duration of BI may have marginal incremental effects on behavioural change [6]. Regarding the counselling method, there is evidence that face-to-face Motivational Interviewing (MI) efficiently decreases alcohol consumption [18], but whether the same effect could be achieved in telephone contacts is still a matter of speculation. Further, self-help materials appear as a promising tool for changing drinking behaviour [19]. In clinical and Internet-based BI such materials provided an effect that added to that of the principal counselling component [20, 21].

In order to contribute to the evidence base of telephone counselling for the consumption of hazardous amounts of alcohol, an individually randomised controlled trial (RCT) was designed at the SAH. The overarching aim of this study is to evaluate the effectiveness of a brief, structured intervention based on self-help followed by one proactive call compared to usual care in modifying drinking patterns. Usual care at the SAH consists of client-driven on-demand counselling with a varying number of proactive and reactive telephone calls based on MI. Secondary purposes of the trial are to compare the effects of the two types of counselling in other health domains such as mental health. In this paper we describe the study protocol of this RCT conducted at the SAH.

## Methods/design

**Fig. 1:**
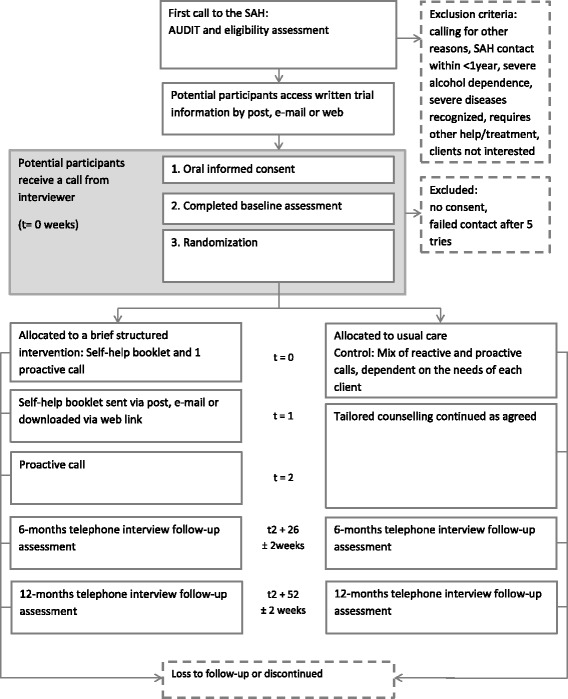
Schematic overview from recruitment to follow-up endpoint of the randomised controlled trial (RCT) at the Swedish National Alcohol Helpline

The study is designed as a superiority, two-group RCT with follow-up at two time points (6 and 12 months). Clients are individually randomised with a 1:1 ratio to brief, structured intervention or usual care using a computer algorithm and allocation concealment. See Fig. 1 for a schematic overview of the trial. We follow the Standard Protocol Items: Recommendations for Interventional Trials (SPIRIT) Checklist for reporting the study protocol, attached as Additional file 1.

### Setting

The trial is carried out at the SAH, a telephone service with national coverage that provides free-of-charge counselling on two to three parallel lines, operating 33 h per week, on weekdays. Written information about the SAH is readily available in health care settings, at the Internet and in social media. The SAH is financed by the Public Health Agency of Sweden and Stockholm County Council (SCC) that also holds the operational responsibility of the service.

In 2015, the SAH received 1849 calls from individuals who were consuming hazardous or harmful amounts of alcohol. There were 640 clients who called for the first time, 58% of whom were men. For first-time callers, the mean age was 48 years for men and 46 years for women (available for *n* = 415), and the mean Alcohol Use Disorder Identification Test (AUDIT) score was 21.1 and 21.5 for men and women, respectively (available for *n* = 407).

### Participants

#### Inclusion criteria

Clients are eligible to participate in the study if they:

1. Are ≥18 years of age, the legal drinking age in Sweden
2. Are first-time callers or callers with a ‘wash out’ period of at least 12 months since their last contact with the SAH
3. Are seeking help for current use of hazardous or harmful amounts of alcohol (identified by a score of ≥6 for women and ≥8 for men on the AUDIT) [22]
4. Are able to speak and read basic Swedish
5. Give informed consent to be randomised into one of the counselling groups and to participate in the follow-up interviews

#### Exclusion criteria

Clients will be excluded from the study if they:

1. At the time of the call exhibit the features of severe alcohol dependence in need of more intense treatment, as identified by SAH counsellors through a cumulative assessment combining a high score on the AUDIT, psychological comorbidities and a history of treatments for alcohol dependence
2. Use illicit drugs
3. Have severe mental illness or suicidal intention
4. Report acute health problems in need of other treatment/medical care

Clients excluded from the study for any of the reasons above, or who wish to terminate participation, will receive counselling according to the SAH current treatment regimen or will be referred to other health care facilities. Concomitant care is permitted during the study period.

#### Trial arms

##### Brief, structured intervention

This intervention includes the delivery to participants of a self-help booklet followed by one proactive call from a counsellor at the SAH. The self-help booklet is designed as a guide to change alcohol use patterns using a cognitive behaviour therapy (CBT) framework. The booklet is developed by the Clinic for Alcohol and Health in Stockholm City, slightly modified for the SAH setting. The structure follows a step-by-step approach; starting from the clients’ initial reflections and motivation to change, proceeding to goal-setting and self-monitoring of their drinking behaviour. In addition, the material contains suggestions on how to practically accomplish behaviour avoidance and resistance skills. The booklet is provided by e-mail, mail or through a password-protected website. After 2 weeks from the dispatching of the booklet, the client is contacted by a proactive call from a counsellor at the SAH which has the scope to assess and guide the use of the material as well as to discuss the client’s progress. After the proactive call, no further contact with the client is initiated by counsellors at the SAH. However, clients are not discouraged from calling the SAH again if they feel the need to do so. If a further contact is initiated by the client, usual counselling is provided. The intervention is delivered according to a protocol and all contacts are logged in a computerised client record which is also used to monitor intervention adherence.

##### Usual care

Usual care provided at the SAH is based on MI, with components of CBT [23, 24]. The counselling aims to: develop resistance skills, promote the clients’ motivation to change and prevent recidivism into the use of hazardous amounts of alcohol. Sessions are tailored to the specific needs of each client such as the severity of their alcohol problems and their readiness to change. In practice, the number and duration of counselling sessions vary and the contact mode can be reactive (client-activated) and/or proactive (counsellor-activated). Clients may request contact with the same counsellor in subsequent calls. Since usual care admits multiple counselling sessions, each building on the outcome of the previous one, all client contacts are registered in a computerised client record. Adherence to the agreed contacts is monitored using information from client records. Since usual care is client-activated, there is no standardised protocol for the counselling.

##### Qualification of counsellors

Counsellors at the SAH are qualified within health care or health promotion and receive training in MI as well as education about the consequences and effects of alcohol use prior to employment. The training consists of a 14-day programme of which six are full days of MI training, distributed over a 4-month period. The MI teaching includes 2.5 days of practical training using recorded interviews with simulated clients and group feedback sessions. The counsellors’ MI performance is evaluated throughout training and employment by coding according to the Motivational Interviewing Treatment Integrity (MITI) Code Version 3.1 [25] conducted at the Motivational Interviewing Coding Laboratory (MIC Lab) at the Karolinska Institutet in Stockholm.

### Outcomes

#### Primary outcome

The primary outcome will be measured at the 6- and 12-month follow-up, through the alcohol screening instrument, AUDIT, validated to identify problematic alcohol use at the lower end of the spectrum, i.e., hazardous and harmful use [26, 27]. AUDIT encompasses three domains: the use of hazardous amounts of alcohol, dependence symptoms and harmful alcohol use. It consists of 10 questions each scored from 0 to 4, thus yielding a maximum scale score of 40. The scale score can be divided into risk levels: ‘Low risk’ (score 0–7 for men and 0–5 for women), ‘Hazardous alcohol use’ (8–15 for men and 6–13 for women), ‘Harmful alcohol use’(16–19 for men and 14–17 for women) and ‘Highly problematic alcohol use’ (≥20 for men and ≥ 18 women) [22].

The primary outcome in this study is defined as a downward shift in AUDIT risk level (yes/no) between baseline and follow-up. The proportion of participants undergoing this shift will be used to aggregate the data.

#### Secondary outcomes

We will address outcomes related to health and behavioural factors including (a) change in depression and anxiety measured using The Mini-International Neuropsychiatric Interview (M.I.N.I.). The outcome is defined as a shift from a M.I.N.I. score indicating depression or generalised anxiety disorder (GAD) to no indication of depression or GAD [28], (b) changes in indicators of general health such as reduction by at least half of sick-leave days and improvements in self-rated health from ‘bad’ or ‘very bad’ to ‘moderate’, ‘good’ or ‘very good’, (c) occurrence of any reported help-seeking for alcohol problems at other treatment providers, such as specialised health care or Alcoholics Anonymous, in the 6- and 12-month intervals.

The M.I.N.I. was developed to accurately detect psychiatric diagnoses according to the *Diagnostic and Statistical Manual of Mental Disorders, version 4* (DSM-IV) and the *International Classification of Diseases, 10th edition* (ICD-10) [28]. The administration time is short and especially suitable for clinical trials and epidemiology studies. The M.I.N.I. is built in modules which can be used separately for rapid screening; this trial employs the modules for depression and GAD.

Self-rated health is used to monitor changes in health over time and has been reported to be a predictor of mortality [29]. The question: ‘How would you rate your overall health’ with the five possible choices ‘very good’, ‘good’, ‘moderate’, ‘bad’, or ‘very bad’ is frequently used within Sweden and the EU.

### Sample size

The sample size estimation is based on the primary outcome, i.e., decrease in AUDIT risk category, in a superiority design. Based on a previous observational study of the SAH [12], we estimate that 30% in the usual care group will make a downward shift in AUDIT risk level. We estimate that 300 participants will be recruited to the study, of which at least 250 participants will be included in the analytical sample (125 in each experimental group). Based on the analytical sample the study will have more than 90% statistical power to detect a Risk Ratio (RR) equal to or greater than 1.7 for the alternative counselling (i.e., 50% cumulative rate of change in the brief, structured intervention group) as statistically significant at the 5% level. For a RR of 1.3 the power will decrease to about 50%.

### Recruitment

The recruitment procedure will be activated when a new client first calls the SAH.

#### Procedure of recruitment

The procedure for participant recruitment was developed in collaboration with responsible staff at the SAH. Before conducting the trial, the feasibility of the procedure was tested in a pilot study.

After assessing eligibility, the counsellors briefly inform eligible clients about the study and ask if they would agree to receive further information. In case of interest, counsellors:

1. Ask in which mode clients wish to receive written information (i.e., e-mail, mail or web)
2. Provide information about the baseline interview and randomisation procedure and ask for the clients’ preferred time of the interview
3. Register contact information and the client’s willingness to participate in the study
4. End the counselling session and terminate the call as soon as possible
5. Send out the required written information

Within five working days, interested clients are contacted by an interviewer, who completes the following sequence of tasks:

1. Ascertains that the client received sufficient information about the study
2. Asks for formal consent to participate to be recorded in the client’s record
3. Conducts the baseline interview
4. Opens a sequentially numbered, sealed envelope containing the results of the randomisation algorithm and communicates the group assignment to the client
5. Schedules the proactive call for all participants randomised to the brief, structured intervention and upon agreement for participants randomised to usual care
6. Informs the research coordinator of the allocation of each participant

The coordinator records the allocation in the study-specific database and creates an electronic prompt for the next step (e.g., proactive call) in the client record at the SAH.

### Randomisation

A computer-generated sequence of serial numbers for up to 1000 participants paired with a random series of allocation numbers (1 for usual care or 2 for the brief, structured intervention) is used to allocate the participants to either trial group. The allocation sequence is based on simple randomisation and is concealed to counsellors, clients and interviewers until the baseline interview is performed. The research coordinator prepares the sequential numbered, sealed envelopes containing a folded note of the group allocation. At the end of the baseline interview, interviewers open the sealed envelope according to the serial number sequence and disclose the allocation, i.e., assign clients to the trial condition.

#### Blindness

Because of the nature of the study, it is impossible to blind the counsellor and the participants concerning the content of the received intervention. However, neither the counsellor nor the participants are aware of the specific effect measures of the study. As the disclosure of the group allocation will take place after recruitment in the study and after the baseline interview, the knowledge of trial group allocation will not influence counsellors in their first contact with the clients. Further, it will not influence the interviewers and the participants at the moment of collecting the baseline information. Moreover, the outcome assessment at follow-up will be conducted by interviewers blinded to the participant’s allocation.

### Data collection

**Fig. 2:**
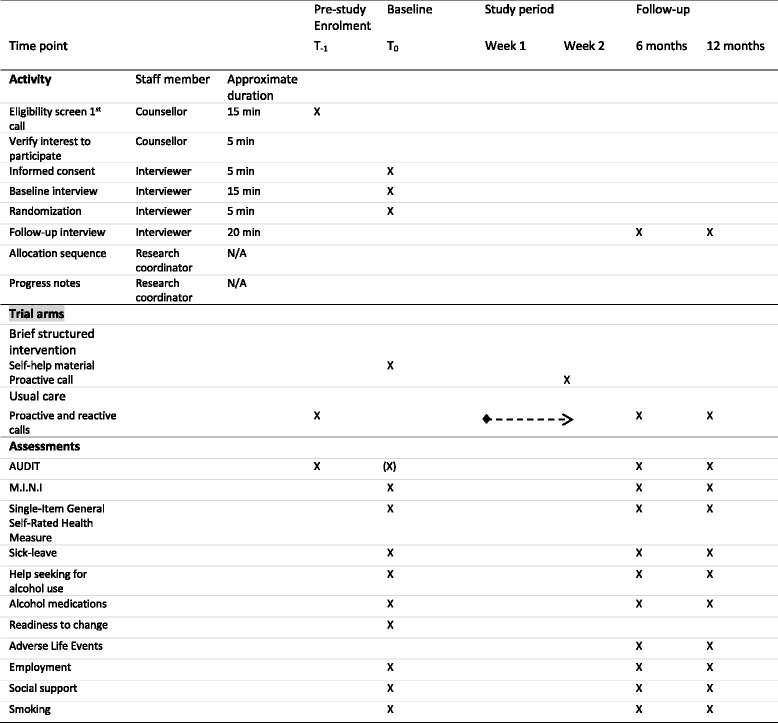
Standard Protocol Items: Recommendations for Interventional Trials (SPIRIT) schedule of enrolment, intervention and assessment, including information of staff members and task duration. *Usual care – tailored counselling mixed approach reactive and proactive calls as agreed. Starts from week 1 or when client wishes to contact the Swedish National Alcohol Helpline (SAH) and continues as needed

The assessment of clients’ characteristics and of the outcome is conducted using a structured questionnaire with closed-ended response alternatives administered via telephone interviews at baseline, 6-month and 12-month follow-up. The questionnaire includes demographics, the AUDIT scale, self-rated health, sick leave, depression or anxiety symptoms (M.I.N.I.) and help-seeking due to problematic alcohol use. Figure 2 displays the process of recruitment, allocation according to the SPIRIT guidelines.

The baseline interview is conducted 5–15 working days after the first call. Follow-up interviews are collected 26 weeks (±2) and 52 weeks (±2) after the baseline interview.

A maximum of five call attempts are made to reach participants at each stage of the data collection, and text messages are additionally used as alerts or to schedule interviews. A research coordinator ensures that participants are followed-up within the appropriate time interval. Data of the process, compliance to the study protocol and adherence of the client will be gathered from the client records kept at the SAH. Available information includes: conduct of proactive and reactive calls as agreed, number of contacts during the study period, and length of each contact.

#### Training of interviewers

Interviewers are not involved in the counselling at the SAH. They receive information about the study and are provided with a quick reference guide containing expressions to use during the interview and a protocol to handle critical events, e.g., expressed suicidal thoughts. Interviewers are trained to assist during three sessions with experienced personnel before conducting any interview on their own.

#### Data security, quality and management

The same rules for data protection and confidentiality are applied for the computerised client record used in the trial as for clinical records in health care. The password-protected record is kept at the SAH, and is used in the everyday management. De-identified information from each participant’s SAH record (baseline AUDIT, duration and number of calls) and data from interviews will be merged into one database, available to investigators in the study team. All employees at the SAH, as well as the personnel in the study team who access personal information, sign a confidentiality agreement upon employment.

Steps involving data management, analysis and interpretation will be carried out by a study team independent both from the funders and from the SAH management. The study team consist of a project leader, a coordinator, a statistician and the interviewers collecting the data. Withdrawal from participation in the trial will instantly be documented in the SAH record and enable participants to contact the SAH for regular counselling. The study process and recruitment process will be discussed in monthly meetings including personnel from the study team and SAH.

Because of the size and nature of the trial, no interim analyses of the intervention’s effects are planned; moreover we do not expect adverse outcomes or significant results before the endpoint. However, loss to follow-up in the two trial conditions is closely monitored as an indication of unfavourable outcome in any group. For the above reasons, no formal data monitoring committee has been assigned.

### Statistical methods

The primary analysis will follow a superiority design applying an intention-to-treat (ITT) approach, i.e., analysing participants based on their random allocation. The alternative hypothesis is that the brief, structured intervention is more effective than usual care in promoting change in a client’s alcohol drinking habits.

#### Statistical analysis

The primary outcome ‘change in AUDIT risk level’ will be analysed as a dichotomous variable where any downward shift (yes/no) from a higher- to a lower-risk level between baseline and follow-up is considered as a clinically meaningful change in drinking habits. The ratio of the cumulative rates of change in the trial groups will constitute the effect measure of the brief, structured intervention versus usual care. General linear models (GLM) will be used to calculate the RR for the outcome event.

Baseline characteristics will be compared between groups in order to detect potential imbalance in predictors of the outcome. If the two groups are not well balanced according to these predictors, multivariable analysis applying logistic regression models will be carried out to estimate odds ratios for the outcome event.

Secondary outcomes (i.e., depression, anxiety, health measures, care seeking) will be analysed using GLM or logistic regression models as described above.

Complementary ‘per-protocol’ and ‘as-treated’ analyses will also be conducted [30]. The ‘per-protocol’ analysis, will only include participants with complete adherence. Complete adherence is defined as: having used the material and received the proactive call (brief, structured intervention); receiving all counselling sessions agreed between the counsellor and the client (usual care). In the ‘as-treated’ analysis, the number of sessions and total duration of the counselling will be considered for each participant, independent of group allocation. These analyses will contribute to the assessment of the effect of the intervention when delivered in standard practice. Possible confounding effects must be carefully monitored in these analyses, since they ignore the random assignment and, therefore, the distribution of baseline risks between participants.

Moderating factors in the analysis include readiness to change, as well as dose-response, i.e., number and length of calls. Further, process data from the records will provide information on the conduct of the proactive calls (adherence).

#### Procedures for missing data

Losses to follow-up will be first and foremost analysed in reference to differential selection. As a primary approach, all available participants will be analysed. As a second step, we will perform sensitivity analyses using ‘worst case scenario’ (loss to follow-up equalised to no change or change to a higher AUDIT category) as well as last observation carried forward (LOCF) in the long-term follow-up. As a third step we will analyse whether missing outcome information can represent cases of at least missing at random (MAR). In this latter case multiple imputation of missing variables data will be conducted, and the results compared with those of the available cases analysis.

## Ethical issues

### Ethical approval

Ethical approval to the study was granted by the Ethical Review Board of Stockholm (DNR 2014/1732-31/5).

#### Information and informed consent

Information about the study is provided at different stages of the recruitment process. Oral information is provided at the initial call to the SAH, followed by the dispatch of written information in Swedish, by mail or e-mail, to interested clients within one week. Additionally, written information is available on the website (alkohollinjen.se/om-alkohollinjen/studier-pa-alkohollinjen/pagaende-studie/).

At the time of the telephone interview, the staff ascertains that clients have received and understood the information. At this point, informed consent is obtained orally and registered in the survey form.

All participants are informed that participation is voluntary and that they can choose to withdraw from the study at any point. The identity of the participants is not disclosed at any time and participants have the possibility to use an alias rather than their actual name during the contacts with both SAH and study staff. All participants are entitled to receive a summary of their registered personal information on a yearly basis.

Due to the inclusion and exclusion criteria and to the nature of the trial, we do not expect any harmful consequence of the counselling in either study arm.

### Dissemination of results

Outcome results will be disseminated in peer-reviewed journals with minimal time from the completion of data collection. Results will be reported separately for the 6-month and the 12-month follow-up. Results will be made available to the contributors and participants of the study, as well as to the funding agency and to the administrators of the SAH through the appropriate websites. No interim analyses are planned.

## Discussion

Telephone helplines constitute a promising approach to the delivery of brief interventions to consumers of hazardous amounts of alcohol, but high-quality studies are needed to determine their effectiveness. Thus, this study is set up as a randomised control trial evaluating the long-term effectiveness of telephone counselling for the use of hazardous amounts of alcohol intended to reach the general population.

The present evaluation will add to previous knowledge [11, 12] by evaluating two separate counselling models delivered at the SAH. The study results will be, first and foremost, used locally in order to further develop the service provided at the SAH. To this end the results will be disseminated promptly to the stakeholders involved in running the helpline. Further, the study will expand the evidence base for counselling models in telephone services on alcohol prevention and treatment, knowledge suitable for use in broader environments.

The principal advantages of the proposed study are the random allocation of study participants, which minimises bias, and the timing of disclosure which does not interfere with data collection at baseline or willingness to participate. Data collection, conducted with personal interviews, will maximise participation and prevent attrition which can be assumed to be large in the target group. Additionally, assessment of the baseline characteristics and of the outcome measures is blinded to allocation and independent from the SAH setting. Incidentally, the data collection and recruitment procedures are quite robust to critical accidents and deviations as they were piloted before the initiation of the trial. In order to monitor the process, data on programme fidelity and participants’ compliance to the intervention will be collected. This information will enable secondary approaches, such as per-protocol and as-treated analyses.

The study also has some limitations. Because of ethical concerns, it was not possible to have a ‘non-treatment’ control group in the SAH setting. The downside of this is the limitation in drawing inference about the effect of the usual care delivered at the SAH.

However, the pragmatic design provides results on the effectiveness and, thus, implications for practical usability of the results. If the brief, structured intervention proves superior to usual care, this would be a strong indication to change to a less labour-intensive method.

Second, blindness at the client and at the counsellor level is not possible, as in many experimental studies based on counselling techniques. This may entail a certain degree of information bias (social desirable answers) in self-reported outcomes. Efforts will be made to keep the potential for bias due to lack of blinding as low as possible, for instance by concealing the study hypotheses and by blinding the interviewers at follow-up as to the individual’s group belonging.

## Trial status

This is the second version of the protocol, modifications have been communicated to the ISRCTN registry. The recruitment of participants is ongoing. Recruitment started on 27 May 2015 and is expected to be completed in December 2017.

## Additional file

Additional file 1: SPIRIT 2013 Checklist: recommended items to address in a clinical trial protocol and related documents. (DOC 122 kb)

## Abbreviations

AUDIT: Alcohol Use Disorders Identification Test
BI: Brief intervention
CBT: Cognitive behavioural therapy
ITT: Intention-to-treat
M.I.N.I.: The Mini-International Neuropsychiatric Interview
RCT: Randomised controlled trial
RR: Risk Ratio
SAH: Swedish National Alcohol Helpline
SEK: Swedish krona
